# The role of prophylactic transfusion on the maternal and fetal outcomes in pregnant women with sickle cell disease: A systematic review and meta-analysis

**DOI:** 10.1097/MD.0000000000039475

**Published:** 2024-09-06

**Authors:** May AlMoshary, Maria Arabdin

**Affiliations:** a Department of Basic Science, College of Medicine, Princess Nourah bint Abdulrahman University, Riyadh, Saudi Arabia; b Qazi Hussain Ahmad Medical Complex, Nowshera Medical College, Nowshera, Khyber Pakhtunkhwa , Pakistan.

**Keywords:** blood transfusion, meta-analysis, sickle cell disease

## Abstract

**Background:**

In the present review, we aimed to synthesize evidence from studies on the safety and effectiveness of prophylactic blood transfusion in pregnant women with sickle cell disease.

**Material and methods:**

To gather relevant information, we conducted systematic electronic searches of databases such as SCOPUS, Medline via PubMed, Web of Science, and Cochrane Central Register of Controlled Trials. We included both retrospective and prospective studies that examined the impact of prophylactic blood transfusions during pregnancy. The collected data were analyzed using Review Manager, version 5.3.

**Results:**

The review included 15 cohort studies. The overall findings indicated a preference for the prophylactic blood transfusion group over the control group across several key parameters. Specifically, the prophylactic group demonstrated lower rates of maternal mortality (odds ratio [OR] = 0.33; 95% confidence interval [CI] = 0.10–1.13; *P* = .08), reduced incidence of vaso-occlusive painful events (OR = 0.31; 95% CI = 0.14–0.73; *P* = .007), fewer pulmonary complications (OR = 0.21; 95% CI = 0.08–0.53; *P* = .001), decreased perinatal mortality (OR = 0.35; 95% CI = 0.17–0.75; *P* = .03), and lower likelihood of preterm birth (OR = 0.67; 95% CI = 0.47–0.96; *P* = .02). Notably, statistically significant heterogeneities were observed in the pooled effect estimates.

**Conclusion:**

The present meta-analysis indicated that prophylactic blood transfusion in pregnant women with sickle cell disease may improve maternal and fetal outcomes. However, substantial variations in the methodology and transfusion protocols among the included studies limited the credibility of the current evidence supporting the routine clinical use of prophylactic transfusion for SCD during pregnancy.

## 1. Introduction

Sickle cell disease (SCD) is one of the most commonly inherited disorders globally and is characterized by diverse clinical severities and potential serious complications.^[1]^ The progression and severity of the disease primarily hinge on the SCD genotype, with hemoglobin (HbS) SS and HbS/β0 exhibiting greater severity, whereas HbSC and HbS/β+ manifested a milder course; however, adverse events have been noted across all subtypes.^[2]^

A sickle point mutation in the *β-globin* gene leads to the production of abnormal sickle-shaped erythrocytes containing sickle HbS, which has lower solubility than normal fetal or adult HbS and is prone to polymerization in the absence of oxygen.^[3]^ These resulting polymers reduce the deformability of erythrocytes and disrupt blood flow in small vessels. This, along with changes in the red blood cell volume and enhanced attachment to the vascular endothelium, initiates inflammation and the pathophysiological mechanisms of blood vessel blockage and red blood cell breakdown.^[2,3]^ These occurrences are especially evident in hypoxic situations, resulting in damage from ischemia-reperfusion, endothelial dysfunction, and damage to multiple organs.^[4]^

Pregnant women with SCD face significant risk, as evidenced by multiple reports highlighting elevated rates of maternal morbidity and mortality, as well as adverse perinatal outcomes such as vaso-occlusive pain episodes, pulmonary complications, preeclampsia, low-birth-weight infants, preterm birth, maternal mortality, and perinatal mortality.^[5–9]^ A recent systematic review and meta-analysis by Oteng-Ntim et al^[3]^ further quantified these risks, indicating that women with SCD are at a heightened risk of experiencing preeclampsia, maternal death, stillbirths, preterm deliveries, and small-for-gestational-age (SGA) newborns.

Prophylactic blood transfusion has been proposed as a strategy to mitigate complications associated with SCD by addressing severe anemia, reducing sickling in both maternal and placental circulation, and improving blood flow and oxygenation.^[10]^ The beneficial effects of transfusion in SCD have been substantiated in various contexts, including preoperative optimization and prophylaxis against stroke.^[11,12]^ However, the role of prophylactic transfusions in SCD during pregnancy has been debated. A previous Cochrane systematic review highlighted the lack of clear evidence regarding the efficacy of prophylactic transfusion in SCD pregnancies, leading to the withdrawal of the corresponding article.^[13]^ Subsequent individual studies have reported conflicting results regarding elective blood transfusion as a prophylactic measure in reducing painful crises, preventing acute chest syndrome, and managing other complications associated with SCD.^[14,15]^

A recent meta-analysis conducted by Malinowski et al^[16]^ showed a correlation between prophylactic blood transfusion in pregnant women with SCD and a reduction in maternal mortality, episodes of vaso-occlusive pain, and pulmonary complications. However, the authors noted that the relatively small number of included studies and their methodological limitations prevented them from making definitive statements regarding the role of prophylactic blood transfusion in SCD pregnancies.^[16]^ As a result, our study aimed to systematically review both the latest updates and previous studies to assess the impact of prophylactic transfusion compared with on-demand transfusion on maternal and fetal outcomes in pregnant women with SCD.

## 2. Materials and methods

This systematic review and meta-analysis was conducted following the guidelines outlined in the Preferred Reporting Items for Systematic Reviews and Meta-Analysis and Meta-Analysis of Observational Studies in Epidemiology statements.^[17,18]^

### 2.1. Inclusion and exclusion criteria

This review incorporated both prospective and retrospective studies that met the following criteria: (1) studies involving pregnant women with SCD; (2) studies evaluating the safety and effectiveness of routine prophylactic blood transfusion in pregnant women with SCD; and (3) In studies comparing prophylactic transfusion with an on-demand transfusion approach, we prioritized the most comprehensive report when multiple reports were available. We excluded non-English studies, reviews, theses, and conference proceedings.

### 2.2. Search strategy and screening

An electronic search was conducted from the beginning of available records until January 2024 in multiple bibliographic databases, including Medline via PubMed, Web of Science, SCOPUS, and the Cochrane Central Register of Controlled Trials. Different combinations of keywords, such as “prophylactic transfusion,” “pregnancy,” “sickle-cell disease,” and “SCD,” were utilized during the search procedure. The identified citations were imported into EndNote 21 to eliminate any duplicates. Afterward, the unique citations were transferred to an Excel spreadsheet and independently evaluated by 2 reviewers. The evaluation process consisted of 2 stages: an initial assessment based on titles and abstracts followed by a comprehensive review of full texts to identify potentially eligible records.

### 2.3. Data extraction and efficacy measures

Data entry and processing were performed using an Excel spreadsheet, where 2 reviewers were tasked with extracting information from the studies included in the analysis. The extracted information covered several areas, including (1) demographic and methodological characteristics of the studies, (2) assessment of bias in the studied populations, (3) specifics of transfusion protocols, and (4) study outcomes. Both reviewers independently gathered data from the included articles and resolved any discrepancies through discussion. The outcomes assessed in studies examining the prophylactic transfusion’s impact on SCD during pregnancy included maternal mortality, perinatal mortality, occurrences of vaso-occlusive painful events, preeclampsia, pulmonary complications, preterm birth, and SGA babies.

### 2.4. Risk-of-bias assessment

The evaluation of the retrieved observational studies’ quality utilized the Newcastle Ottawa Scale. This assessment considered the selection of study groups, comparability between groups, and the ascertainment of exposure or outcome. Each study was assigned a cumulative quality score ranging from 1 to 9, and based on this score, studies were classified into categories of high risk of bias (scores 1–3), moderate risk of bias (scores 4–6), and low risk of bias (scores 7–9).^[19]^

### 2.5. Data synthesis

We performed a paired comparison meta-analysis utilizing the Review Manager, version 5.3, software designed for Windows. The combination of dichotomous data was done as odds ratios (ORs) accompanied by 95% confidence intervals (CIs) employing the Mantel-Haenszel method. The heterogeneity of the combined effect estimates was evaluated using the I^2^ statistic alongside its respective *P* values. Due to significant heterogeneity among the studies included, we opted for a random-effects model. All reported *P* values were 2 sided, and statistical significance was considered for *P* values < .05.

## 3. Results

### 3.1. Literature search results

**Figure 1.**
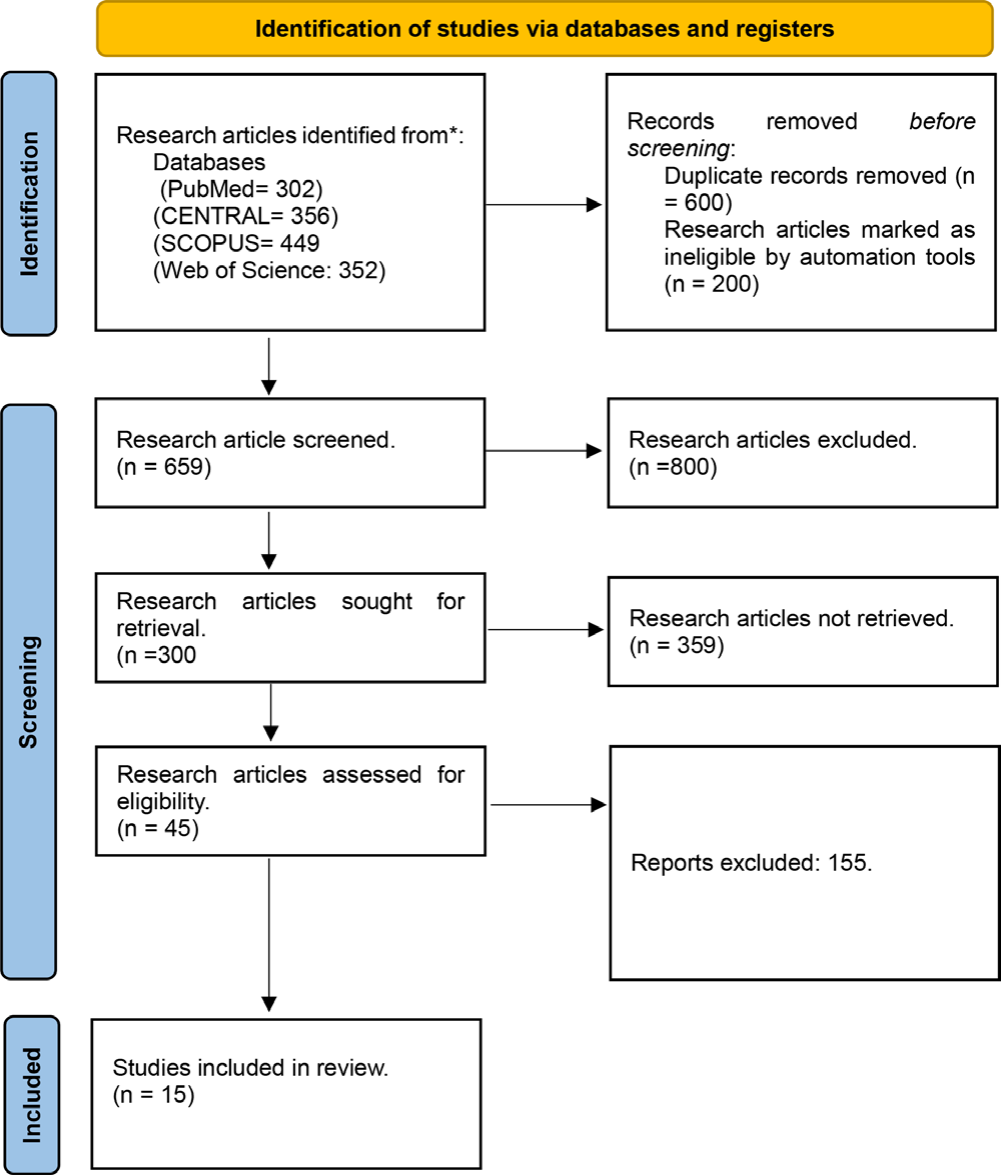
Screening and selection of included studies by PRISMA guidelines. PRISMA = Preferred Reporting Items for Systematic Reviews and Meta-Analysis.

The initial literature search resulted in 1459 unique citations after removing duplicates. After title and abstract screening, 45 potentially eligible records were identified for full evaluation. Ultimately, 15 studies were included in this systematic review and meta-analysis (931 patients in the prophylactic group and 854 patients in the control group) (Fig. 1).

### 3.2. Characteristics of studies

The present review comprised 15 quasi-randomized trials.^[10,14,15,20–31]^ All quasi-trials were retrospective cohort studies, except for the study by Morrison et al,^[31]^ which was a prospective cohort study with a historical control group. The sample sizes of the included studies ranged from 24 to 571 patients. Notably, there were significant variations in the protocols for prophylactic blood transfusion for SCD among the included studies. Additionally, studies have reported conflicting results regarding the efficacy and safety of prophylactic blood transfusion for SCD. Tables 1 and 2 provide detailed characteristics of the included studies.

**Table 1:** Characteristics of studies included.

Cohort studies							
Author, year	Country setting	Single versus multicenter	Groups	Sample size	Years of study	Inclusion criteria	Conclusion
Benites et al, 2016^[20]^	The University of Campinas Hospital	Single-center retrospective cohort	Administering either prophylactic erythrocytapheresis or manual exchange transfusion during the 28th week of pregnancy	10	June 1994 and August 2014	SCD	The research showed a significant decrease in negative outcomes among patients who received preventive transfusions, likely indicating improved maternal and fetal health conditions. This also aligned with the better vitality indicators observed in newborns.
			Transfusions are given only when necessary due to acute complications, or in some cases, no transfusions are required at all	14			
Asma et al, 2015^[15]^	Turkey	Single-center retrospective cohort	Prophylactic blood transfusion	24	2000–2013	SCD	This study has shown that administering pRBCX during pregnancy is a viable and safe approach to prevent complications.
			Indicated transfusion	13			
Gilli et al, 2007^[14]^	Brazil	Single-center retrospective cohort	Administering prophylactic erythrocytapheresis transfusions at the start of the third trimester	14	1994–2004	SCD	The research indicates that erythrocytapheresis transfusions offer advantages for women with SCD in the 3rd trimester.
			Providing basic preventive transfusions solely based on the severity of SCD, without any other specific indication	17			
Howard et al, 1995^[10]^	United Kingdom Hospitals in England and Wales	Multicenter retrospective cohort	Prophylactic blood transfusion	29	1991–1993	SCD	It is advised to implement a strategy of exchanging transfusions for all women with homozygous SCD (HbSS) starting from the 28th week of pregnancy to minimize the chances of maternal complications during the third trimester and postpartum period.
			Indicated transfusion	34			
El-Shafei et al, 1995^[22]^	Bahrain Ministry of Health Hospitals	Single-center mixed retrospective and prospective cohorts	Prophylactic blood transfusion	244	Prospective: 1988–1994 Retrospective: 1986–1988	SCD	A cautious approach to blood transfusion can be implemented without putting at risk the health of the fetus or mother, and it also offers the added advantage of decreasing the occurrence of transfusion-related crises and other associated complications.
			Indicated transfusion	327			
Koshy et al, 1991^[23]^	United States	Single-center retrospective cohort	Prophylactic transfusion, beginning at the early weeks of pregnancy	36	1986–1990	SCD	
			Transfusion only	35			
Morrison et al, 1991^[24]^	United States	Single-center retrospective cohort with controls	Performing a partial prophylactic exchange transfusion early in prenatal care	103	January 1981 to December 1990	SCD	The findings suggest an advantage of using this approach in treating pregnant patients with SCD.
			Indicated transfusion on demand	28			
Tuck et al, 1987^[26]^	United Kingdom	Multicenter retrospective cohort	Prophylactic blood transfusion	36	1978–1984 (prophylactic transfusion) 1975–1981 (untransfused)	SCD	The data show no significant difference in fetal or maternal outcome between those patients who were transfused prophylactically and those who were not.
			Indicated transfusion	26			
Cunningham et al, 1983^[25]^	United States	Single-center retrospective cohort with historical controls	Prophylactic blood transfusion	54	1973–1982 (PT) 1955–1972 (ODT)	SCD	Prophylactic blood transfusions significantly decrease maternal complications and the risk of perinatal death.
			Indicated transfusion	54			
Miller et al, 1981^[21]^	United States	Single-center retrospective cohort with controls	Prophylactic blood transfusion	25	1978–1980 (prophylactic transfusion) 1974–1979 (on-demand transfusion) Historical controls	SCD	The exchange transfusion may best be reserved as a treatment modality when infection, crises, or symptomatic anemia occurs in gravidae with sickle hemoglobinopathies.
			Indicated transfusion	36			
Morrison et al, 1976^[31]^	United States	Single-center prospective cohort, matched retrospectively with historical controls	Prophylactic blood transfusion	36	1970–1974 1965–1970	SCD	Administering prophylactic partial exchange transfusions to pregnant individuals with severe sickle cell hemoglobinopathies can be advantageous.
			Indicated transfusion	29			
Ribeil et al, 2018^[27]^	France	Multicenter retrospective cohort study	Prophylactic transfusion strategy	191 cases	- 2005–2010 2011–2014	SCD	It is safe to combine targeted transfusion with preventive at-home nocturnal oxygen therapy to lower the need for transfusions and the risks associated with them.
Yilmaz Baran et al, 2020^[28]^	Turkey	Single-center, retrospective, cross-sectional study	pRBCX	27	January 2012 and June 2019	SCD	It shows that by reducing adverse fetomaternal outcomes, pRBCX in SCD positively impacts the course of pregnancy.
				19			
Ngo et al, 2010^[29]^	France	Retrospective case-control study	Prophylactic blood transfusion	SCD = 128	January 2019	SCD	The prevalence of SCD remains the same, and as severe complication, it affects pregnant women despite prophylactic blood transfusion.
				AA phenotype = 128			
Sousa et al, 2022^[30]^	Brazil	Multicenter, retrospective, cross-sectional study	Prophylactic transfusions	40	January 1, 2010, to December 31, 2019	SCD	The current study’s findings regarding the decreased incidence of fetal death during pregnancy in women who received more transfusions during their pregnancies.

HbSS = hemoglobin SS, ODT = on demand transfusion, pRBCX = prophylactic red blood cell exchange, PT = prophylactic transfusion, SCD = sickle cell disease.

**Table 2:** Summary of management and transfusion protocols.

Cohort studies									
Author, year	Prophylactic transfusion					On-demand transfusion			
	Type of transfusion	Timing of initiation, GA (wk)	MUT	Proportion requiring ODT (%)	Initial targets subsequent triggers	Reason for transfusion	Timing of initiation, GA (wk)	MUT	Proportion requiring ODT (%)
Asma et al, 2015^[15]^	Continuous-flow apheresis	Variable	6.2 ± 0.6	16.7%	Target: HbS < 30% Trigger: Hb < 7.0 mg/dL, Hct = 27%	Simple transfusion: Hb < 7.0 mg/dL, leukocytosis in the absence of infection	1st and 3rd trimesters	NA	15%
Gilli et al, 2007^[14]^	Erythrocytapheresis	28th	8.14 ± 4.31	NA	Target: NA Trigger: NA	Acute complications of SCD	NA	3.7 ± 2.7	NA
Howard et al, 1995^[10]^	Transfusion depending on the severity of anemia	1st or 3rd trimester	NA	NA	Target: NA Trigger: NA	Complications of SCD	NA	NA	26%
El-Shafei et al, 1995^[22]^	Partial transfusion	NA	NA	60.7%	Target: NA Trigger: NA	Low Hb < 60 mg/dL, sickle crisis	NA	NA	29.4%
Koshy et al, 1991^[23]^	Simple or partial exchange transfusion	1st transfusion	12	NA	Target: HbS < 35% Trigger: NA	Before C/S, severe anemia	NA	4	74%
Morrison et al, 1991^[24]^	Partial exchange transfusion	19.4	NA	NA	Target: HbA > 50% Trigger: HbA < 20%	Severe anemia	NA	NA	58%
Tuck et al, 1987^[26]^	Simple or partial exchange transfusion	Depending on HbSS	12	NA	Target: Hb = 11.0 mg/dL Trigger: NA	NA	NA	NA	NA
Cunningham et al, 1983^[25]^	Simple transfusion or partial exchange transfusion	20th week of gestation	13	NA	Target: HbA = 50% Trigger: HbA < 40%	Hb < 7.0 mg/dL	NA	NA	NA
Miller et al, 1981^[21]^	Partial exchange transfusion	23rd	13	NA	Target: NA Trigger: HbA < 25%	Hb < 7.0 mg/dL	NA	3.7	NA
Morrison et al, 1976^[31]^	Partial exchange transfusion	28th	6	5.6%	Target: HbA = 40% Trigger: HbA < 20%	Severe anemia	NA	1	38%
Ribeil et al, 2018^[27]^	Simple transfusion	28th	5	NA	Target: HbA ≥ 25% Trigger: Hct ≤ 25%, HbA ≤ 20%	Severe anemia	NA	NA	NA
Yilmaz Baran et al, 2020^[28]^	Partial exchange transfusion	3rd trimester	7	NA	Target: HbS ≤ 30% Trigger: Hb ≤ 9 mg/dL	Anemia, vaso-occlusion crisis	NR	3	2%
Ngo et al, 2010^[29]^	Partial exchange transfusion	3rd trimester	NA	NA	Target: Hb = 9 mg/dL, HbS ≤ 40% Trigger: Hb ≤ 9 mg/dL	Severe anemia, crisis	NA	23	2.1%
Sousa et al, 2022^[30]^	Partial exchange transfusion	3rd trimester	NA	NA	Target: Hb = 10 g/dL, HbS ≤ 50% Trigger: Hb = 8.0 mg/dL	Severe anemia, crisis	NA	NA	40.7%

C/S = cesarian section, GA = gestational age, Hb = hemoglobin, HbS = hemoglobin, HbSS = hemoglobin SS, Hct = hematocrit, MUT = mutation, NA = not available, NR = not reported, ODT = on demand transfusion, SCD = sickle cell disease, SHbA = adult hemoglobin.

### 3.3. Risk-of-bias assessment

In the included studies, risk of bias ranged from moderate to high according to the Newcastle Ottawa Scale. Specifically, all studies exhibited a high risk of bias in the comparability and follow-up domains. A summary of the risk-of-bias assessment is provided in Table S1, Supplemental Digital Content, http://links.lww.com/MD/N452.

### 3.4. Outcomes

#### 3.4.1. Maternal outcomes

**Figure 2.**
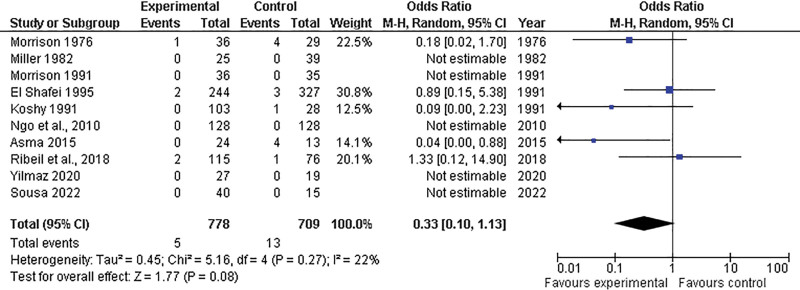
Forest plot depicting maternal mortality comparison between prophylactic transfusion and control groups. CI = confidence interval, M-H = Mantel-Haenszel Test, df = Degrees of freedom.

Ten of the included studies provided data on maternal mortality, comparing the prophylactic (n = 778 patients) and control (n = 709 patients) groups. The overall effect estimates favored the prophylactic group over the control group (OR = 0.33; 95% CI = 0.10–1.13; *P* = .08), with no significant heterogeneity identified (*P* = .2) (Fig. 2).

**Figure 3.**
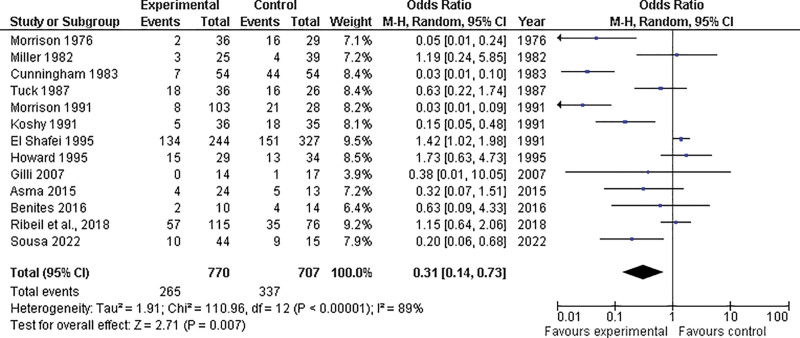
Forest plot illustrating the comparison of vaso-occlusive pain events between the prophylactic transfusion and control groups. CI = confidence interval, M-H = Mantel-Haenszel Test, df = Degrees of freedom.

On the other hand, 13 studies reported the incidence of vaso-occlusive painful events, showing that the prophylactic group had lower rates than the control group (OR = 0.31; 95% CI = 0.14–0.73; *P* = .007). However, significant heterogeneity was detected (*P* < .00001; I^2^ = 89%) (Fig. 3).

**Figure 4.**
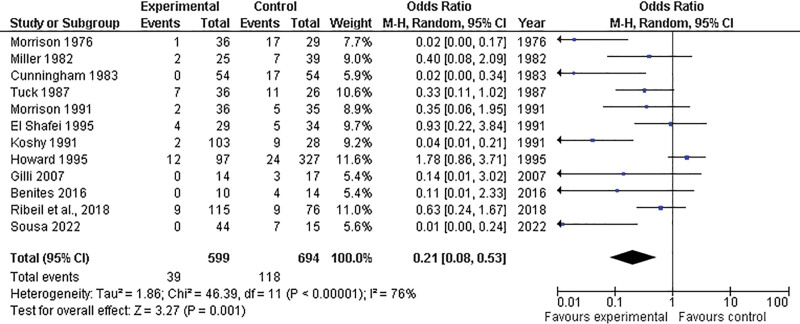
Forest plot illustrating the comparison of pulmonary complications between the prophylactic transfusion and control groups. CI = confidence interval, M-H = Mantel-Haenszel Test, df = Degrees of freedom.

Similarly, the prophylactic group exhibited fewer pulmonary complications than the control group (OR = 0.21; 95% CI = 0.08–0.53; *P* = .001), although there was significant heterogeneity (*P* < .01; I^2^ = 76%) (Fig. 4).

**Figure 5.**
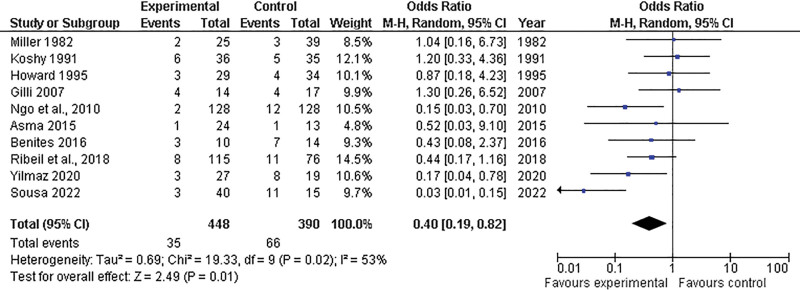
Forest plot depicting the comparison of preeclampsia between the prophylactic transfusion and control groups. CI = confidence interval, M-H = Mantel-Haenszel Test, df = Degrees of freedom.

In contrast, the prophylactic group showed a significant advantage over the control group in terms of preeclampsia (OR = 0.40; 95% CI = 0.19–0.82; *P* = .02) (Fig. 5).

#### 3.4.2. Fetal outcomes

**Figure 6.**
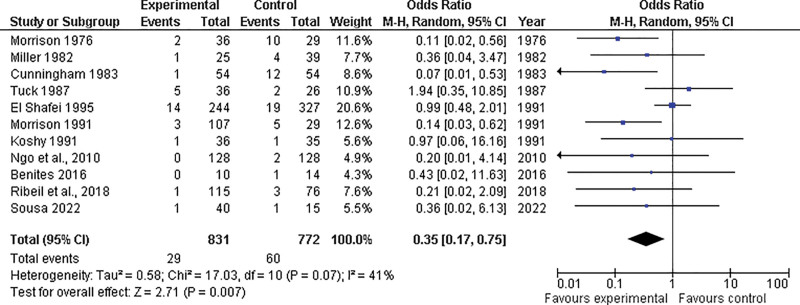
Forest plots illustrating the disparity in perinatal mortality between the prophylactic and control groups. CI = confidence interval, M-H = Mantel-Haenszel Test, df = Degrees of freedom.

Regarding fetal outcomes, 11 studies included in our analysis reported differences in perinatal mortality between the prophylactic (n = 831 patients) and control (n = 772 patients) groups. The overall effect estimates favored the prophylactic group over the control group (OR = 0.35; 95% CI = 0.17–0.75; *P* = .03); however, there was moderate heterogeneity in the pooled effect estimate (*P* = .07; I^2^ = 41%) (Fig. 6).

Similarly, 9 studies reported on the effect of prophylactic transfusion on the incidence of preterm birth, with the overall effect estimates favoring the prophylactic group over the control group (OR = 0.67; 95% CI = 0.47–0.96; *P* = .02); no significant heterogeneity was identified (*P* = .13). In contrast, the overall effect estimate did not favor the prophylactic group over the control group in terms of the incidence of SGA newborns (OR = 0.58; 95% CI = 0.55–1.32; *P* = .46).

## 4. Discussion

The existing literature presents divergent findings regarding the effectiveness and safety of prophylactic transfusion in pregnant women with SCD. In this systematic review and meta-analysis, prophylactic blood transfusion demonstrated a statistically significant reduction in both maternal and perinatal mortality compared with on-demand transfusion. Additionally, the prophylactic transfusion group exhibited a significantly lower incidence of vaso-occlusive painful events, pulmonary complications, and preterm birth. However, there was a significant advantage observed for the prophylactic group concerning the incidence of preeclampsia and SGA newborns. It is important to note that there were statistically significant heterogeneities in most of the pooled effect estimates.

SCD is one of the most prevalent hereditary disorders, characterized by severe adverse events in its severe forms. It is well documented that pregnancy is a major risk factor for serious maternal complications in SCD patients, with recent studies indicating significantly elevated risks of maternal mortality, preeclampsia, and painful vaso-occlusive events in pregnant women with SCD.^[3]^

However, there remains a lack of consensus regarding the most effective transfusion strategy for managing pregnancy-associated adverse events in patients with SCD. In our meta-analysis, we found that prophylactic blood transfusion led to a statistically significant reduction in maternal mortality, incidence of vaso-occlusive painful events, pulmonary complications, and reduction in preeclampsia compared with on-demand transfusion. This aligns with the findings of Malinowski et al,^[16]^ who reported similar reductions in maternal mortality, vaso-occlusive pain episodes, pulmonary complications, pulmonary embolism, and pyelonephritis associated with prophylactic transfusion.

Likewise, a recent systematic review indicated a statistically significant reduction in maternal mortality and vaso-occlusive pain episodes following prophylactic blood transfusion.^[32]^ However, it is important to note the contrasting results from the only available randomized controlled trial in the literature that reported no statistically significant improvements in maternal outcomes with prophylactic transfusion, except for the incidence of painful vaso-occlusive events.^[33]^ The ability of this trial to detect significant results is questionable owing to its small sample size and high risk of bias.

Additionally, pregnancy has a notable impact on fetal outcomes in patients with SCD. Existing evidence indicates that pregnancy significantly increases the risk of perinatal mortality, preterm birth, and intrauterine growth restriction in this population.^[34,35]^ Our meta-analysis revealed that the prophylactic blood transfusion group had a significantly lower perinatal mortality rate and preterm birth incidence. However, there was no significant advantage observed for the prophylactic group in terms of the incidence of SGA newborns.

Consistent with our findings, Jackson et al^[32]^ reported that the majority of available data indicated lower perinatal mortality rates in the group that received prophylactic blood transfusions. Malinowski et al^[16]^ reported similar results regarding improved fetal outcomes associated with prophylactic transfusion.

The current systematic review and meta-analysis had several strengths. The review adhered to the recommendations outlined in the Cochrane Handbook and the Preferred Reporting Items for Systematic Reviews and Meta-Analysis statement, ensuring a rigorous and transparent methodology. Furthermore, controlled studies were exclusively included to mitigate the risk of methodological bias.

However, it is important to acknowledge the limitations of this study. Significant inconsistencies were observed in the pooled effect estimates, likely because of wide variations in the characteristics of the study populations, designs, and transfusion protocols. Additionally, the methodological quality of the included studies ranged from low to moderate, which may have affected the overall quality of the evidence presented in this review.

## 5. Conclusion

In conclusion, the findings of the present meta-analysis suggest that prophylactic blood transfusion in pregnant women with SCD may lead to improved maternal and fetal outcomes. The prophylactic group showed lower rates of mortality, painful vaso-occlusive events, pulmonary complications, preeclampsia, perinatal mortality, and preterm births. However, it is crucial to note that there were significant variations in the methodology and transfusion protocols across the included studies, which resulted in considerable statistical heterogeneity in the pooled effect estimates.

Current evidence appears to be insufficient to definitively support the clinical decision to routinely use prophylactic transfusion over symptomatic transfusion for SCD during pregnancy. Further well-designed studies are warranted to provide more robust evidence.

## Author contributions

**Conceptualization:** May AlMoshary.

**Data curation:** May AlMoshary, Maria Arabdin.

**Formal analysis:** May AlMoshary, Maria Arabdin.

**Funding acquisition:** May AlMoshary.

**Investigation:** May AlMoshary.

**Methodology:** May AlMoshary.

**Project administration:** Maria Arabdin.

**Resources:** Maria Arabdin.

**Software:** Maria Arabdin.

**Supervision:** Maria Arabdin.

**Validation:** Maria Arabdin.

**Visualization:** Maria Arabdin.

**Writing – original draft:** Maria Arabdin.

**Writing – review & editing:** Maria Arabdin.

## Supplementary Material


