# Supplementary material for: The role of prophylactic transfusion on the maternal and fetal outcomes in pregnant women with sickle cell disease: A systematic review and meta-analysis
Source: Medicine (Baltimore). 2024 Sep 6;103(36):e39475. doi: 10.1097/MD.0000000000039475 (PMC12431739; doi:10.1097/MD.0000000000039475)
Supplement: Supplementary file 1 [file medi-103-e39475-s001.docx]

**Supplementary table 1: Risk of Bias Assessment for Included Studies**

|  | | | | | | | | | | |
| --- | --- | --- | --- | --- | --- | --- | --- | --- | --- | --- |
|  | **COHORT STUDIES** | | | | | | | | | |
|  |  | **Outcomes** | | | **Comparability** | | **Selection** | | | **Author (Year)** |
| **Risk of Bias**  **1-3: High**  **4-6: Moderate**  **7-9: Low** | **Total /8** | **Adequacy of follow-up (loss)** | **Appropriate Follow-up (length)** | **Assessment of outcomes** | **Control for confounders** | **Comparability of Groups on 2ryrisk factors** | **Outcome of interest Not present at study start** | **Ascertainment of Exposure** | **Representativeness of exposed cohort** |  |
| High | 2 |  |  | ***** |  |  |  |  | ***** | **Benites et al., 2016** |
| High | 2 |  |  | ***** |  |  |  |  | ***** | **Asma et al., 2015** |
| - Moderate | 4 |  |  | ***** | ***** |  |  | ***** | ***** | **Gilli et al., 2007** |
| Moderate | 4 | ***** |  | ***** |  |  |  | ***** | ***** | **Howard et al., 1995** |
| High | 3 | ***** |  |  |  |  |  | ***** | ***** | **El-shafei et al., 1995** |
| High | 2 |  |  | ***** |  |  |  |  | ***** | **Koshy et al., 1991** |
| - High | 3 |  |  | ***** |  |  |  | ***** | ***** | **Morrison et al.,1991** |
| High | 1 |  |  |  |  |  |  |  | ***** | **Tuck et al., 1987** |
| High | 3 |  |  | ***** |  |  |  | ***** | ***** | **Cunningham et al., 1983** |
| High | 3 |  |  | ***** |  |  |  | ***** | ***** | **Miller et al., 1981** |
| High | 3 |  |  | ***** |  |  |  | ***** | ***** | **Morrison 1976** |
| Moderate | 5 | * | * | ***** |  |  |  | ***** | ***** | **Ngo et al., 2010** |
| Moderate | 4 |  | * | ***** |  |  |  | ***** | ***** | **Ribeil et al., 2018** |
| Moderate | 4 |  | * | ***** |  |  |  | ***** | ***** | **Sousa et al., 2022** |
| High | 3 | * |  | ***** |  |  |  |  | ***** | **Yilmaz et al., 2020** |
